# *In Vitro* Antimycobacterial Activity of Pakistani Beri Honey Using BACTEC MGIT 960

**DOI:** 10.1155/2014/490589

**Published:** 2014-10-07

**Authors:** Abdul Hannan, Saira Munir, Muhammad Usman Arshad, Nabila Bashir

**Affiliations:** University of Health Sciences, Lahore, Pakistan

## Abstract

*Background*. Tuberculosis (TB) is a chronic bacterial disease. *Mycobacterium tuberculosis*, being the leading member of the MTB complex, is the main cause of tuberculosis worldwide. Tuberculosis is managed with combination of drugs: streptomycin, isoniazid, rifampicin, ethambutol, and pyrazinamide. Over the recent past years resistance against first line antituberculous drugs has emerged rapidly throughout the world resulting in MDR strains. The new threat in the management of MDR-TB is the development of resistance against second line drugs: aminoglycosides, polypeptides, fluoroquinolones, and thioamides. Multidrug resistant (MDR) and extensively drug resistant TB (XDR) strains have become a major concern to control TB particularly in the developing countries. The need of the hour is to look for new modalities having antimycobacterial activity. Honey has been well known for its antibacterial activity. We intended to explore its antimycobacterial activity against MDR-TB. *Objective*. The objective of this study was to determine whether Pakistani Beri honey has any antimycobacterial activity. *Method*. The study was conducted in the Department of Microbiology, University of Health Sciences, Lahore. Clinical isolates (*n* = 21) of MDR-MTB were evaluated for their susceptibility to Beri honey. The isolates were provided, courtesy of Pakistan Medical Research Council. These isolates were identified by MTBc ID test (Becton & Dickinson) and further tested for their antimycobacterial activity using Beri honey. The honey was tested at the following concentrations (v/v): 1%, 2%, 3%, 4%, and 5% in MGIT 960. Growth controls were also inoculated with each isolate (growth control has no concentration of honey, only containing growth of isolate). *Results*. MDR-TB isolates (*n* = 21) were tested; 3 (14%) isolates were susceptible at 1% v/v honey, while at 2% v/v of honey 18 (86%) isolates were found to be susceptible. All the 21 isolates (*n* = 21) were susceptible at 3% v/v of honey. *Conclusion*. The present study clearly demonstrates that Pakistani Beri honey possesses significant antimycobacterial activity *in vitro*. The antimycobacterial activity of Pakistani Beri honey may, therefore, be exploited in an appropriate mouse model.

## 1. Introduction 

Tuberculosis is a chronic bacterial infectious disease (“as discussed by Daniel [1]”) that has become a disease of major public health concern globally. Tuberculosis is being treated with combination of drugs for many decades. The regimens that use only single drug result in the rapid development of resistance and hence treatment failure (“as discussed by Wang et al. [2]”). The new threat in the management of TB is the emergence of resistance to standard anti-TB drugs that leads to multidrug resistance (“as discussed by Lawn and Wilkinson [3]”). Multidrug resistance (MDR) has become a major concern to control TB particularly in the developing countries (“as discussed by Cohn et al. [4]”). MDR-TB is an emerging problem of global magnitude, with higher mortality rates than sensitive drugs for TB (“as discussed by Diraa et al. [5]”). It is estimated that about 450,000 new MDR-TB cases occur every year (“as discussed by Thaver and Ogunbanjo [6]”). Conventional drugs usually provide effective antibiotic therapy for bacterial infections. However, there is an increasing problem of antibiotic resistance and a continued need for new solutions. Herbs, plants, and other natural substances are used in folk medicine as a treatment for bacterial infections worldwide. Honey is a natural product, present almost everywhere, affordable, and acceptable to everyone. According to our knowledge there is no evidence that has been reported of any resistance against this natural product. Honey is increasingly becoming part of medicine and it has been approved as therapeutic agent for leg ulcers, diabetic foot ulcers, burns, skin graft donor sites, and surgical wounds particularly where conventional treatment fails. This natural remedy is still grossly underutilized and it is a time for researcher to explore its therapeutic potential on multitude of purposes (“as discussed by Zumla and Lulat [7]”). Van Ketel was the first who reported the antibacterial properties in 1892 and Dustmann mentioned this work in 1979 (“as discussed by Dustmann [8]”). The word inhibine was coined by Dold in 1937 after an extensive study on the antimicrobial effects of honey. Subsequent research identified inhibine as hydrogen peroxide generated by an enzyme from bee gland. Since then various other studies have been published on this subject which confirmed the antibacterial potential of honey (“as discussed by Efem et al. [9]”). The antimicrobial activity of honey is mainly attributed to its acidity, high osmolarity, hydrogen peroxide generated by glucose oxidase (bee origin), and nonperoxide factors (plant origin) (“as discussed by Molan [10]”). The glucose oxidase is a heat and light sensitive enzyme, secreted from the hypopharyngeal gland of the bee and found to be in an inactive state in undiluted honey (“as discussed by Dustmann [8]”). This enzyme converts water and sugar into hydrogen peroxide when honey is diluted (“as discussed by Franchini et al. [11]”). Therefore, this makes undiluted honey an ideal therapeutic agent as exudates from wound surface dilute it and consequently enhance its antibacterial activity. More than one hundred botanical species from which European unifloral honeys can be produced are identified and surprisingly no two honeys are completely the same (“as discussed by Oddo and Bogdanov [12]”). Honey, amazingly, on one hand inhibits more than sixty different species of pathogenic bacteria and on the other hand enhances the growth of normal flora of gastrointestinal tract (“as discussed by Olofsson and Vasquez [13]”).

Considering the multiple strategies adopted by honey to defeat pathogenic organisms with direct effect and indirectly, orally taken honey may serve as an ideal candidate for future prevention and treatment of diseases. Honey produced from each plant species and from each geographical area has its own color, flavour, aroma, texture, granularity, viscosity, antibacterial properties, and prebiotics composition (“as discussed by Molan [10]”), since Pakistan produces large quantity of most valuable Beri honey which is comparatively darker in colour as compared to other Pakistani honeys (“as discussed by Khan et al. [14]”). Dark coloured honeys such as manuka (New Zealand), chestnut (Canadian), jelly bush (Australia), and avocado honey are famous for their high level of antibacterial activity (“as discussed by Gheldof et al. [15]”). The role of honey as topical therapeutic agent for skin and other pathogenic agents has been well established (“as discussed by Okeniyi et al. [16]”). In this study we have evaluated antimycobacterial activity of honey.

## 2. Materials and Methods

### 2.1. Setting

This study was conducted at the Department of Microbiology, University of Health Sciences (UHS), Lahore. The study was based on the determination of antimycobacterial activity of Beri honey against clinical isolates of MDR* Mycobacterium tuberculosis*. The duration of study was from May 2011 to May 2012.

#### 2.1.1. Bacterial Isolates

A total of 21 MDR-MTB isolates grown on LJ medium were tested for antimycobacterial activity. These isolates were kindly provided by Pakistan Medical Research Council TB Centre, Mayo Hospital Lahore.

#### 2.1.2. Pakistani Beri Honey

Pakistani Beri honey was provided by Punjab honey farms, Lahore.

#### 2.1.3. Identification of MTB Isolates

The isolates were initially identified by their morphological characteristics using Ziehl-Neelsen staining technique. The isolates were further confirmed by MTBc ID (*Mycobacterium tuberculosis* complex identification) test kit (Becton & Dickinson, USA).

#### 2.1.4. Subculture of MTB Isolates

The isolates were subcultured in middlebrook 7H9 broth and were incubated in MGIT 960. The positive declared vials by the instrument were further subjected to identification of MTB and antimycobacterial activity of Beri honey. The positive vials checked for the confirmation of AFB presence by Ziehl-Neelsen staining and MTBc ID test.

#### 2.1.5. Identification of MTB Complex by MTBc ID Kit

Identification of MTB complex was done by MTBc identification kit provided by Becton & Dickinson. The BD MTBc ID kit is a rapid immunochromatographic assay for the qualitative detection of* Mycobacterium tuberculosis* complex antigen MPT64 from AFB smear positive vial. 0.1 mL of sample was added into the sample well of the labelled test kit. Reading was taken after 10 minutes; in case of positive results, a pink to red line was appeared in test and control areas which shows the presence of MPT64 antigen in the sample.

#### 2.1.6. Confirmation of MDR-MTB Isolates

*Drug Susceptibility Testing (DST).* All the isolates were recognized to be MDR by subjecting them to standard concentration of streptomycin (332 *μ*g), isoniazid (33.2 *μ*g), ethambutol (1660 *μ*g), and rifampin (332 *μ*g) using MGIT 960 according to protocols in MGIT procedure manual.

*Antimycobacterial Activity of Beri Honey.* The antimycobacterial activity of Beri honey was tested with 1% to 5% concentration of Beri honey.

*Inoculation of MGIT Tubes.* MGIT tubes were labelled with date of inoculation, concentration of honey, and growth control. The tubes were inoculated in MGIT 960 and were monitored till the instrument declared growth control positive.

*Growth Control Inoculation Procedure.* For growth control inoculation, 0.5 McFarland suspension of isolate was diluted to 1 : 100 (100 microliters of isolate suspension from 0.5 McFarland and 9.9 mL of normal saline). From this diluted suspension 0.5 mL was added in the growth control MGIT vial and 0.8 mL of PANTA was also added. There was no honey in growth control vials. The tubes were monitored for 42 days according to standard protocol before declaring them negative.

## 3. Results

At 1% concentration of honey more than 18 isolates out of 21 were found resistant. At 2% only 03 isolates out of 21 were found resistant and 18 were susceptible; however, at 3%, 4%, and 5% concentrations of honey all isolates were found susceptible (Table 1).

## 4. Discussion

The use of honey as a traditional remedy for microbial infections dates back to ancient times (“as discussed by Molan [17]”). Research has been conducted on manuka honey, which is found to be effective against several human pathogens, including* Escherichia coli*,* Enterobacter aerogenes*,* Salmonella typhimurium*, and* Staphylococcus aureus*. Laboratory studies conducted by Allen et al. in 2001 revealed that the honey is effective against methicillin-resistant* S. aureus* (MRSA), haemolytic streptococci, and vancomycin-resistant enterococci (VRE). In the current study, we have used Beri honey that is locally produced and this study demonstrated that Beri honey inhibited all MDR strains of MTB used in this study (*n* = 21) at a concentration ranging from 1% to 5%. These results are probably being presented for the first time against MDR-TB using 7H9 Middlebrook broth dilution technique and BACTEC MGIT 960. Due to MDR mycobacteria being common and emergence of extensively drug resistant (XDR) TB, there is strong need to evaluate natural products like honey which may provide some headway in managing MDR-TB. MGIT 960 being a new technique was used in this study, to evaluate* in vitro* activity of honey. 21 isolates of MDR-MTB were tested for their antimycobacterial activity, 3 strains were inhibited at 1% v/v honey, another 15 strains were inhibited at 2% v/v honey, and the remaining 3 strains were inhibited at 3% v/v honey. The cumulative susceptibility at 2% v/v honey was 86% while 100% inhibition was achieved at 3% v/v honey. Previously Asadi-Pooya et al. in 2003 studied Avicenna honey and they demonstrated that Avicenna honey inhibits MTB strains at a concentration of 10% to 20%. This work was done on solid LJ medium. In contrast to this study we have used MGIT 960 that is more sensitive and rapid detection method than conventional LJ medium (“as discussed by Asadi-Pooya et al. [18]”). They only studied 2 strains; we are evaluating much larger number (*n* = 21) as per our knowledge; this study might have tested or evaluated the highest numbers so far. Also our honey used, that is, Beri honey, has a much superior* in vitro* performance. Another important thing is that our study was conducted at a more sophisticated automatic instrument that gives much faster results whereas the other study mentioned used LJ medium slopes. The results with this latter normally take around 3 weeks' incubation, whereas BACTEC MGIT 960 gives results in 10 days. Finally, the therapeutic potential of honey holds great promise. In future employment of Beri honey should be considered as an effective treatment modality for MDR-MTB. It is suggested that this study may be expanded on to evaluation of larger number of strains as well as in some appropriate animal model. The outcome may be expected to be ultimately beneficial for clinical trials.

## 5. Conclusion

Currently, the emerging antimicrobial resistance trends in MTB are a serious challenge. Thus, the findings of this study, together with previous study of Asadi-Pooya et al. in 2003, show that honey offers promise as an effective anti-MTB or MDR-MTB treatment.

## Figures and Tables

**Table 1:** Antimycobacterial activity of honey against MTB isolates (*n* = 21) at different concentrations.

Concentration	1%	2%	3%	4%	5%
Susceptible (−)	03 (14%)	18 (86%)	21 (100%)	21 (100%)	21 (100%)
Resistant (+)	18 (86%)	03 (14%)	NIL	NIL	NIL

Total	21 (100%)	21 (100%)	21 (100%)	21 (100%)	21 (100%)

Key:

(+): growth positive (resistant to honey);

(−): growth negative (susceptible to honey).
